# Risk factor analysis of omicron patients with mental health problems in the Fangcang shelter hospital based on psychiatric drug intervention during the COVID-19 pandemic in Shanghai, China

**DOI:** 10.3389/fpsyt.2023.1100849

**Published:** 2023-02-13

**Authors:** Ping Yu, Xiaolan Bian, Zhihui Xie, Xu Wang, Xujing Zhang, Zhidong Gu, Zhitao Yang, Feng Jing, Weiyu Qiu, Jingsheng Lin, Jie Tang, Chen Huang, Yibo Zhang, Ying Chen, Zongfeng Zhang, Yufang Bi, Hanbing Shang, Erzhen Chen

**Affiliations:** ^1^Department of Pharmacy, Ruijin Hospital, Shanghai Jiao Tong University School of Medicine, Shanghai, China; ^2^Department of Medical Affairs, Ruijin Hospital, Shanghai Jiao Tong University School of Medicine, Shanghai, China; ^3^Department of Asset Management, Ruijin Hospital, Shanghai Jiao Tong University School of Medicine, Shanghai, China; ^4^Department of Foreign Cooperation and Development, Ruijin Hospital, Shanghai Jiao Tong University School of Medicine, Shanghai, China; ^5^Ruijin-Hainan Hospital, Shanghai Jiao Tong University School of Medicine, Bo’ao Research Hospital, Qionghai, Hainan, China; ^6^Department of Emergency, Ruijin Hospital, Shanghai Jiao Tong University School of Medicine, Shanghai, China; ^7^Department of Disciplinary Development and Planning, Ruijin Hospital, Shanghai Jiao Tong University School of Medicine, Shanghai, China; ^8^Department of Hospital-infection Control, Ruijin Hospital, Shanghai Jiao Tong University School of Medicine, Shanghai, China; ^9^Department of Psychiatry, Ningbo Kangning Hospital, Ningbo, Zhejiang, China; ^10^Department of Endocrine and Metabolic Diseases, Ruijin Hospital, Shanghai Jiao Tong University School of Medicine, Shanghai, China; ^11^Department of Neurosurgery, Ruijin Hospital, Shanghai Jiao Tong University School of Medicine, Shanghai, China

**Keywords:** COVID-19, mental health, psychiatric drugs intervention, Fangcang shelter hospital, public health

## Abstract

**Backgrounds:**

The widespread coronavirus disease 2019 (COVID-19) outbreak impacted the mental health of infected patients admitted to Fangcang shelter hospital a large-scale, temporary structure converted from existing public venues to isolate patients with mild or moderate symptoms of COVID-19 infection.

**Objective:**

This study aimed to investigate the risk factors of the infected patients from a new pharmacological perspective based on psychiatric drug consumption rather than questionnaires for the first time.

**Methods:**

We summarised the medical information and analysed the prevalence proportion, characteristics, and the related risk factors of omicron variants infected patients in the Fangcang Shelter Hospital of the National Exhibition and Convention Center (Shanghai) from 9 April 2022 to 31 May 2022.

**Results:**

In this study, 6,218 individuals at 3.57% of all admitted patients in the Fangcang shelter were collected suffering from mental health problems in severe conditions including schizophrenia, depression, insomnia, and anxiety who needed psychiatric drug intervention. In the group, 97.44% experienced their first prescription of psychiatric drugs and had no diagnosed historical psychiatric diseases. Further analysis indicated that female sex, no vaccination, older age, longer hospitalization time, and more comorbidities were independent risk factors for the drug-intervened patients.

**Conclusion:**

This is the first study to analyse the mental health problems of omicron variants infected patients hospitalised in Fangcang shelter hospitals. The research demonstrated the necessity of potential mental and psychological service development in Fangcang shelters during the COVID-19 pandemic and other public emergency responses.

## 1. Introduction

The new coronavirus disease 2019 (COVID-19) has become a global public health emergency which was declared a concern by the World Health Organization (WHO) (1). The omicron variant of the severe acute respiratory syndrome coronavirus 2 (SARS-CoV-2) caused the disease to evolve into a serious epidemic outbreak; it spread quickly around Shanghai in March 2022, with more than 60,000 confirmed positive infected patients by 31 May 2022. The government implemented efficient strategies, such as quick isolation, screening, and rebuilding Fangcang shelter hospital, to protect public health in a timely manner, prevent the spread of the virus, and manage infected people (2).

Fangcang shelter hospital, one of the effective countermeasures, played a powerful role in preventing and controlling (3) which were first implemented as a novel public health concept during the Hubei COVID-19 medical rescue in 2020 (4). Fangcang shelter hospitals concept were originally brought from the military field hospitals as the temporary medical facilities for public health emergencies control. These structures were mostly converted from stadiums, exhibition halls, and other public venues supplying medical services such as infection isolation, disease monitoring and treatment, food supply, and social activities provision. Fangcang shelter hospitals were constructed to supply service for the infected patients with mild or moderate symptoms who are isolated from their families or communities. In the first COVID-19 pandemic of Wuhan, Fangcang shelter hospitals showed have been proven to be the most effective and timely way to prevent virus transmission and control the infection outbreak rapidly (5, 6). These experiences supplied a certain basis for the controlling subsequent infections outbreak. During the COVID-19 pandemic, Shanghai quickly constructed large scale Fangcang shelter hospitals to response to the omicron variant of the COVID-19 pandemic.

The National Exhibition and Convention Center (Shanghai) Fangcang hospital was the largest one designed to accommodate 46,872 beds, which received 174,308 infections from 9 April 2022 to 31 May 2022, and all the infected patients were cured to discharge or transferred to designated hospitals. Such a large number of infected patients required comprehensive attention.

Evidence shows that the occurrence of major public health events like severe acute respiratory syndrome (7, 8) and Ebola virus disease caused not only physical implications but also mental health problems (9). Similarly, people of varied backgrounds affected by COVID-19 reportedly suffered from a burden of psychological problems (10, 11). The impact of COVID-19 on patients’ psychology has caused an international concern (12). Based on questionnaires, previous studies in Wuhan Fangcang shelter hospitals reported that many admitted patients faced anxiety, depression, insomnia, perceived stress, post-traumatic stress symptoms, and so on (13, 14).

Compared with the Wuhan variant of SARS-Cov-2, omicron variants in Shanghai had lower mortality or severity rate. However, it caused a larger-scale pandemic with higher rate of infection, faster spread, and stronger stealthiness which greatly influenced people’s daily life. Before being admitted to the Fangcang shelter hospitals, the normal life of infected patients was disrupted due to the prolonged home quarantine and imposed lockdown of the government. Mental health outcomes, including anxiety, depression, and sleep disorders, were reported to exist extensively among individuals because of inadequate information, life supplies, fears of infection, and boredom (15, 16).

After being admitted, infected patients experienced the temporarily built public environment, which was quite different from the usual hospitals, such as excessive noise, interfering light, and decreased privacy. Further, they had to suffer from the feeling of being separated from family and adapting to a new and strange environment. Some of them often got bothered and felt a lack of hope due to uncertain treatment or outcomes, although their symptoms were mild and moderate (15). Fangcang shelter hospitals could only supply basic medical care and treatment for infected patients with a history of diseases. The facilities were not as comprehensive in meeting patients’ personalised medical needs as the traditional hospitals due to limited medical drugs and instruments. In addition, the family members of infected patients might have had a high risk of infection. These unstable factors could impact the mental health of infected patients and stimulate psychiatric problems of anxiety, depression, or sleep disorders.

Studies have reported different proportions of infected people facing mental health problems in Fangcang shelter hospitals. Patients with severe mental health problems, including psychiatric or psychological disorders, need certain interventions, while most infected persons with mild symptoms could recover autonomously without intervention. All previous studies reported high morbidity of mental health problems in Fangcang shelter hospitals such as the Wuhan Fangcang, based on a questionnaire or assessment scales and analysis with limited data and samples. As a new variant, the characteristics of omicron variant are very different with an unprecedented outbreak in Shanghai. Characters of infected patients might vary as the relatively common accepted vaccination. However, little efforts have been denoted to the mental health problems of omicron variant of SARS-CoV-2 infected people in Fangcang shelter hospitals. This is important since it can help us better understand omicron infection and find the appropriate solution to deal with it. On the other hand, all previous studies are based on a questionnaire or assessment scales with the advantages of economy, easy to operate, and cost-saving. However, the study based on a questionnaire or assessment scales has a strong subjectivity and possesses inevitably defects. First, questionnaire survey is difficult to design comprehensively. Second, the low recovery rate will affect its representativeness. Third, the quality of the acquired information cannot be guaranteed. When respondents fill in the questionnaire, they may give an estimated answer or avoid the essential things, which affects the accuracy of information. Currently, adopting a pharmacological approach according to the drug consumption is more objective. What’s more, serious mental health problems might cause prolonged effect on the patient’s later life or work, or even irreversible. Additionally, the potential risk factors for infected patients with mental health problems in severe conditions proposed by the reported articles were not comprehensive enough. This study aims to evaluate the mental health outcomes of COVID-19 infected patients in Fangcang shelter hospitals according to psychiatric drug consumption and analyse the associated potential risk factors. The infected individual facing mental health challenges was analyzed from a pharmacological perspective based on drug intervention, which has not been reported previously. This study could provide some evidence of the necessity of timely mental health services for targeted populations in Fangcang management shelter hospitals and policy development during the COVID-19 epidemic.

## 2. Methods

The National Exhibition and Convention Centre of Shanghai Fangcang Shelter Hospital were constructed as a temporary medical building for the admission and hospitalisation of infected patients with moderate and mild COVID-19 symptoms. It received 174,308 infected patients from 9 April 2022 to 31 May 2022. The infected patients were cured to discharge or transferred to a designated hospital for treatment with severe symptoms. The information of infected patients who used the drugs as listed (risperidone, olanzapine, quetiapine, paroxetine, sertraline, venlafaxine, flupentixol-melitracen, escitalopram oxalate, zolpidem tartrate, estazolam) was collected as the drug intervention group. Patients diagnosed of schizophrenia were mainly prescribed with risperidone, olanzapine and quetiapine. For depression diagnosis, patients were prescribed with paroxetine, sertraline, venlafaxine, flupentixol-melitracen or escitalopram oxalate according their individual specific symptom. Patients with insomnia were prescribed with zolpidem. And patients with symptoms of anxiety or sleep disorders were intervened with estazolam. The information was integrated when the infected individual used different drugs were classified listed as schizophrenia, depression, insomnia, anxiety or sleep disorder according to the symptom severity from severe to mild. A total of 6,218 individuals treated with the list drugs in the Fangcang shelter hospital were processed. Simultaneously, information of a corresponding comparable control group of 30,000 infected patients who has no listed psychiatric drug intervention was randomly drawn out based on the number of patients in the drug intervention group.

### 2.1. Statistical analysis

All the data were analysed using SPSS version 22 (IBM, Armonk, NY, United States) or GraphPad Prism version 8.0.0 (GraphPad Software, San Diego, CA, United States). Continuous data of hospitalized time for normal variables were quantitatively analysed and presented as mean ± standard deviation. The univariate analysis to study the affected factors was performed using the chi-squared (*χ*^2^) test. All factors with *p* < 0.05 in the univariate analyses were included in the multivariate analysis. The multivariate logistic regression analyses were performed to identify the independent factors using stepwise variable selection. A *p* < 0.05 was considered statistically significant.

## 3. Results

### 3.1. Prevalence of infected patients requiring psychiatric drugs intervention

**Figure 1:**
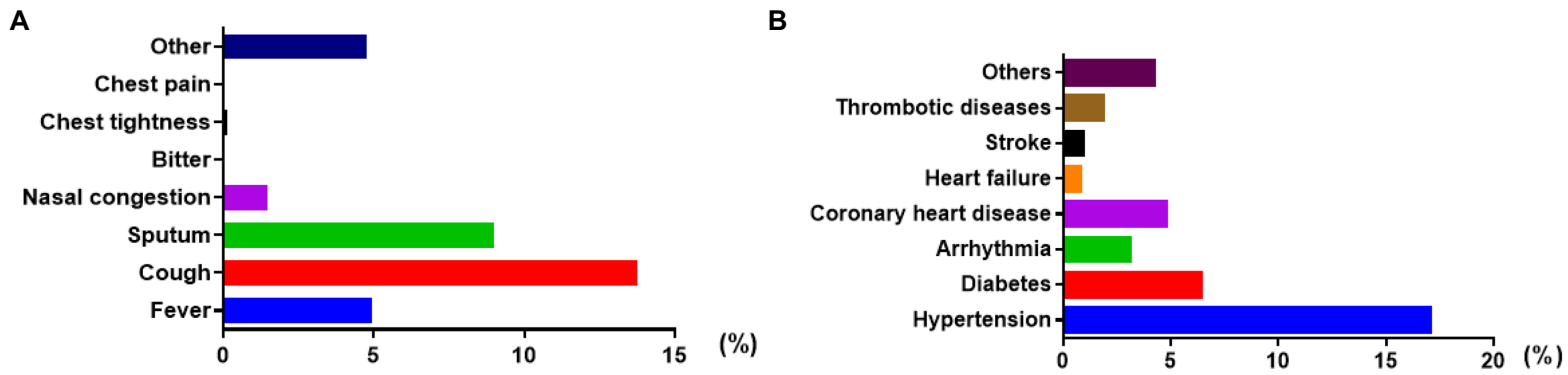
General characteristics of physical symptoms and distribution of comorbidities in infected patients treated with associated psychiatric drugs. **(A)** proportion of COVID-19 symptoms. **(B)** proportion of comorbidities.

A total of 6,218 infected persons who used associated psychiatric drugs were included in the study, making up 3.57% of all the admissions (6,218 of 174,308 infected patients) in the National Convention and Exhibition Centre of Shanghai Fangcang Shelter Hospital of China from 9 April 2022 to 31 May 2022. Among the patients whose data were collected, 3.20% needed drugs to treat schizophrenia, such as risperidone, olanzapine, and quetiapine, with 3.07% being prescribed these drugs for the first time. Further, 1.88% needed drugs to control depression, such as paroxetine, sertraline, venlafaxine, flupentixol-melitracen, and escitalopram. 54.76% needed zolpidem to treat insomnia, with 53.51% having no such previous prescriptions. Likewise, 40.16% needed estazolam to treat anxiety and sleep disorders, with 38.10% being prescribed these drugs for the first time. 3.46% had a history of psychiatric disease (see Table 1). The most common symptom in the group of infected patients associated with COVID-19 was cough, at 13.77% (844 of 6,128 infected patients intervened with psychiatric drugs), followed by subsequent sputum and fever at 8.99% (551 of 6,128) and 4.96% (304 of 6,128), respectively, shown separately in Figure 1A. As shown in Figure 1B, the top three current comorbidities were hypertension at 17.02% (1,043 of 6,128 infected patients intervened with psychiatric drugs), diabetes at 6.38% (391 of 6,128), and coronary disease at 4.80% (294 of 6,128).

**Table 1:** The prevalence of infected patients needing administration of associated psychiatric drugs.

psychiatric drugs	*n* (%)	diagnosis	First prescription (*n*, %)
risperidone	5 (0.08)	schizophrenia	191 (3.07)
olanzapine	34 (0.55)		
quetiapine	160 (2.57)		
paroxetine	32 (0.51)	depression	116 (1.87)
sertraline	27 (0.43)		
venlafaxine	21 (0.34)		
flupentixol-melitracen	31 (0.50)		
escitalopram	6 (0.10)		
zolpidem	3,405 (54.76)	insomnia	3,327 (53.51)
estazolam	2,497 (40.16)	anxiety or sleep disorder	2,369 (38.10)

### 3.2. Patient characteristics

The demographic and clinical characteristics of the treated patients with mental health problems in severe conditions who needed drug intervention were analysed. Similar information of 30,000 patients with no psychiatric drug intervention was randomly drawn out as the comparable control group according to the number of patients in the intervention group. For the group with mental health problems intervened using psychiatric drugs, the prevalence was high among patients who are females (51.57%, 3,160), older (18–44 years, 25.90%, 1,587; 45–59 years, 31.74%, 1,945; ≥60 years, 43.15%, 2,644), unmarried (single, 4,382, 71.51%; divorced, 160, 2.61%; widowed, 127, 2.07%), unvaccinated (2,099, 34.25%), and had more comorbidities (1,848, 13.84%; ≥2,638, 10.41%). The intervention group had a longer hospitalisation time of 9.98 days compared with the control group of 7.29 days. Additional details of data among different groups are shown in Table 2. Statistic results indicated the related factors of infected patients in the psychiatric drug intervention group, including sex, age, marital status, occupation, hospitalisation time, number of concomitant physical symptoms of COVID-19, vaccination, and number of comorbidities based on covariates with *p* < 0.05.

**Table 2:** Characteristics of the sample in different groups.

Characteristic	No drug intervention	drug intervention	*χ* ^2^	Value of *p*
*Sex*, *n* (%)			237.512	<0.0001
Female	12,066 (79.25)	3,160 (20.75)		
Male	17,934 (85.43)	3,058 (14.57)		
*Age*, *n* (%)			3387.812	<0.0001
<=18	1,518 (97.31)	42 (2.69)		
18–44	15,366 (90.64)	1,587 (9.36)		
45–59	9,158 (82.48)	1945 (17.52)		
≥60	3,958 (59.95)	2,644 (50.05)		
*Marital status*, *n* (%)			439.503	<0.0001
Single	17,614 (80.08)	4,382 (19.92)		
Married	11,338 (87.98)	1,549 (12.02)		
Divorced	762 (82.65)	160 (17.35)		
Widowed	286 (69.24)	127 (30.75)		
*Occupation*, *n* (%)			2038.747	<0.0001
self-employed individual	721 (84.82)	129 (15.18)		
worker	4,713 (87.78)	656 (12.22)		
farmer	1,286 (82.65)	270 (17.35)		
business manager	10,481 (83.32)	2098 (16.68)		
retire (leave) personnel	741 (87.18)	109 (12.82)		
jobless people	892 (82.9)	184 (17.1)		
student	1,371 (96.08)	56 (3.92)		
office clerk	4,263 (88.46)	556 (11.54)		
professionals	1,084 (87.7)	152 (12.3)		
freelance	1804 (87.62)	255 (12.38)		
others	2,644 (60.13)	1753 (39.87)		
*Vaccination times*, *n* (%)			277.638	<0.0001
0	7,163 (77.34)	2099 (22.66)		
≥1	22,837 (84.72)	4,119 (15.28)		
*Co-symptoms*, *n* (%)			9.963	0.0189
0	24,035 (82.61)	5,061 (17.39)		
1	2,840 (84.5)	521 (15.5)		
2	2,143 (83.71)	417 (16.29)		
≥3	982 (81.77)	219 (18.23)		
*Comorbidities*, *n* (%)			674.175	<0.0001
0	26,521 (84.86)	4,732 (15.14)		
1	2,155 (71.76)	848 (28.24)		
≥2	1,324 (67.48)	638 (32.52)		
*Hospitalized time*, *n* (Mean ± SD)			*F*	Value of *p*
	30,000 (7.29 ± 3.13)	6,218 (9.98 ± 4.36)	3270.477	<0.0001

### 3.3. Risk factors for infected patients with psychosomatic problems needing drug intervention

The risk factors for infected patients with mental health problems needing psychiatric drug intervention were analysed and presented in Table 3. Sex and vaccination were analysed as categorical variables, while age, hospitalisation time, and comorbidities were analysed as continuous variables. The results of multivariate logistic regression analyses showed that female sex (odds ratio [OR], 1.502; 95% confidence interval [CI] 1.414 to 1.596; *p* < 0.0001), older age (OR, 2.2331; 95% CI, 2.146 to 2.321; *p* < 0.0001), longer hospitalisation time (OR, 1.186; 95% CI, 1.177 to 1.195; *p* < 0.0001), no vaccination (OR, 1.217; 95% CI, 1.139 to 1.301; *p* < 0.0001), and more comorbidities (OR, 1.106; 95% CI, 1.060 to 1.153; *p* < 0.0001) were independent risk factors for infected persons hospitalised in the Fangcang shelter with mental health problems in severe conditions needing psychiatric drug intervention.

**Table 3:** Multivariate logistic regression analysis of risk factors influencing psychiatric drug use.

Variables	B	SE	Walds	Value of *p*	OR (95% CL)
Sex (female/male Ref)	0.407	0.031	175.155	<0.0001	1.502 (1.414 to 1.596)
Age (≥60/45–59/19–44/≤18 Ref)	0.803	0.020	1612.943	<0.0001	2.231 (2.146 to 2.321)
Hospitalized time	0.171	0.004	1783.959	<0.0001	1.186 (1.177 to 1.195)
Vaccination (0/≥1 Ref)	0.197	0.034	33.894	<0.0001	1.217 (1.139 to 1.301)
Comorbidities (≥2/1/0 Ref)	0.100	0.022	21.548	<0.0001	1.106 (1.060 to 1.153)

B, Partial regression weight; SE, Standard error.

## 4. Discussion

To our knowledge, this is the first study analysing mental health problems of COVID-19 infected patients hospitalised in Fangcang shelter hospitals from a new perspective based on psychiatric drug intervention. This study aimed to evaluate the characteristics of COVID-19 infected patients with mental health in Fangcang shelter hospitals according to psychiatric drug consumption, analyse the associated potential risk factors, and eventually explore the necessity of developing mental health services timely for targeted populations in Fangcang management shelter hospitals and policy development during the COVID-19 epidemic. A previous study indicated a more severe level of mental health problems in the Fangcang shelter than the norm. However, severe mental health situations requiring drug interventions have not been previously reported. In this study, we found that 3.57% of all admitted infected patients in the Fangcang shelter hospital needed antipsychotic drugs intervention to treat mental health problems, including schizophrenia, depression, insomnia, and anxiety or sleep disorder. This indicated a more severe situation of mental health of the COVID-19 infected patients admitted to Fangcang shelter hospitals. Among them, 96.54% had no medical history of psychiatric diseases before. Further study revealed that female sex, no vaccination, older age, longer hospitalisation time, and more comorbidities were independent risk factors for hospitalised infected patients with mental health problems in severe conditions needing drug intervention. Though intervention of mental health problem involved in various aspects, it’s an intuitional way to analyze the information according to the psychiatric drugs which has not been reported previously. Understanding the situation of psychiatric drug consumption provided a new insight into the mental health impact of the COVID-19 pandemic on the patients with confirmed positive infected patients in Fangcang shelter hospitals. This may provide evidence for the necessity of potential development of an improvement of psychiatry service for COVID-19 infected patients in Fangcang shelter hospitals or norms during the occurrence of public health emergencies.

Public emergency events could generally cause mental health problems. Globally, the WHO estimated that a disaster resulted in diverse mental health problems for 30–50% of the population (17). Since 30 January 2020, the COVID-19 pandemic has been declared a worldwide public health emergency by the WHO, which was considered an international concern (18). Numerous studies reported that people who experienced COVID-19 infection were at high risk of having mental health challenges such as anxiety, depression, sleep disorder, and even post-traumatic stress disorder. The prevalence of mental health problems existed in confirmed or suspected patients and the general public during the COVID-19 pandemic, while patients with COVID-19 had a higher risk of mental health problems than others (19).

Infected people admitted to Fangcang shelters needed psychiatric drug intervention, which might have had serious consequences, since they faced mental health problems that could not be alleviated *via* any other measures. The current study was generally based on questionnaires, while no study has been reported from the drug intervention perspective. For infected patients diagnosed with serious psychiatric symptoms, such as uncontrolled anxiety or insomnia, the corresponding drugs would be needed (14, 20). In this study, we collected data on all the infected patients who used related psychiatric drugs. These patients may face with severe mental health problem which could lead to prolonged influence for their later life. Results showed that most infected patients facing sleep disorders or anxiety were treated with zolpidem at 54.76% and estazolam at 40.16%. Others were diagnosed with schizophrenia at 3.20% using risperidone, olanzapine, and quetiapine, and depression at 1.88% using paroxetine, sertraline, venlafaxine, flupentixol-melitracen, and escitalopram. A small percentage (3.46%) of infected patients also had basic psychiatric diseases. However, Hao et al. found that psychiatric patients suffered worse physical health and were more susceptible to psychiatric illnesses, such as post-traumatic stress disorder, severe insomnia, depression, anxiety, and stress (21). Further, 96.54% had no diagnosed history of psychiatric disease and 5.08% of cases needed definite antidepressant or anti-schizophrenia drugs, which confirmed the severity of negative mental health impact on infected persons admitted to the Fangcang shelter during the COVID-19 epidemic.

Public health emergencies were the initial and immediate reasons for people experienced psychiatric or psychological health problems. People suffered from irrational nervousness or were scared that the omicron variant of SARS-CoV-2 would cause a life-threatening epidemic disease based on the acknowledgment of the virus in Wuhan (22), while receiving little information on the omicron variant in Shanghai at the beginning of its outbreak. Although it is reported that omicron variant caused significantly lower hospitalisation incidence, shorter hospitalisation time, and less severe admission and fatality rate than any other variants, the risk perception of people was aroused due to the extremely wide eruption of multiple mutations of the omicron variant which resulted in its significant immune escape and unprecedented rapid spread (23, 24).

In addition, large numbers of infected patients have been placed into a longer quarantine isolation or social distancing to control the transmission of the epidemic before admission, which was identified to cause a high risk of mental health problems (16, 25, 26). Unlike the traditional hospital, the large-scale Fangcang shelter hospitals were mostly temporary structures with limited medical conditions and a lack of expected care because of insufficient healthcare workers (13, 27). Infected persons struggled with lifestyle changes and could not adapt to hospital life in the new environment. Patients admitted to Fangcang shelter hospitals may have experienced mental health problems such as loneliness, anger, anxiety, depression, insomnia due to separation from family, perception of uncertain physical discomfort, fear of bad prognosis or uncertain recovery, worry about family members’ infection risk, as well as exposure to negative media coverage. All of these were sources of severe mental health problems for admitted patients, which might have ultimately negatively affected their life quality and social function (14, 28, 29).

Our multivariate logistic regression analysis revealed that female sex was a risk factor for infected patients with severe mental health problems needing drug intervention. The result was in line with a few empirical studies on higher susceptibility and prevalence of mental health problems in women compared with men (30, 31). The COVID-19 pandemic has been reported to have a significantly bad impact on aged individuals, mainly owing to social isolation and health concerns (32–34). Older patients admitted to the Fangcang shelter were more likely to have been living alone and have felt sad when medical care or personalised medicine for their accompanied basic physical diseases was not satisfactory as in general hospitals (35). Thus, our results also showed that older age and more comorbidities are independent risk factors for psychological issues among infected patients as previous reported research (36, 37). Longer hospitalisation time was found to be another independent factor related to the use of psychiatric drugs. Patients may experience mental health problems owing to the uncertain outcome of the infection, anxiety about neighbours’ discharge, and prolonged separation from family. Also, the worse mental health condition could delay the recovery of COVID-19 symptoms owing to the interaction between the two outcomes.

Vaccination with one or two doses has been proven to significantly reduce the severity rate of COVID-19 and protect against hospitalisation and mortality (38). Clinical studies report that booster dose vaccination reduces the symptomatic disease to mild and significant chances of recovery (39, 40). The knowledge of reported information could comfort people’s anxiety to a certain degree. Thus, vaccination may indirectly positively affect the mental situation of patients admitted to the Fangcang shelter by reducing the risk of severe COVID-19. According to the statistics, the mental situation of the infected patients without vaccination was worser than that of patients with vaccination. Our study found that vaccination served as a protection from mental health problems resulting from infected patients which was in accordance with previous studies (41).

## 5. Limitations

This study analysed a large sample of infected persons with severe mental health problems based on the use of psychiatric drugs in the Fangcang shelter. However, the study is lack of the overall mental health situation of infected persons which can influence the analysis results. Questionnaires to evaluate the overall mental health situation are required for further investigation. This study was performed only in the National Convention and Exhibition Center (Shanghai) Fangcang hospital and is lack of representativeness. As temporal structures, the Fangcang shelter hospitals differ in the environment and level of medical care, and this could not be accounted for since our study was not multinational or multicenter. The long-term mental health outcomes of COVID-19 infected patients or the effectiveness of psychiatric drug interventions in mitigating these outcomes need to be investigate as well. Therefore, more related data among infected persons in other Fangcang shelter hospitals need to be collected and analysed. The mental health research in our study was conducted in a short time frame, and follow-up in future longitudinal studies is also needed. The conclusion of this study was analyzed and conducted based on the perceptual information of psychopharmacotherapy. Besides drug therapy, intervention modes of mental health also include psychotherapy, behavior therapy, health education and so on, which are needed to be explored by more studies. Other potential risk factors need further investigation because of the temporary emergency program with limited information collection, which may have influenced the results such as preexisting mental health conditions or social support.

## 6. Conclusion

This study identified the prevalence and characteristics of omicron variants infected patients with mental health problems in severe conditions using psychiatric drugs and analysed the risk factors among these individuals in the Fangcang shelter hospitals. Among them, most experienced sleep disorder or anxiety, needing zolpidem and estazolam intervention. Others needed drugs to treat schizophrenia or control depression symptoms. Female sex, older age, presence of more comorbidities, and longer hospitalisation time were independent risk factors. We also concluded that vaccination had a protective correlation with the mental health of these infected patients. Our findings provided in-depth consideration about the mental health problems of omicron variants infected patients in the Fangcang shelter hospitals, and demonstrated the necessity of intervention service development on public mental health to reduce the negative psychological impact of infected patients in Fangcang shelter hospitals during the COVID-19 pandemic and other public emergency responses.

## Data availability statement

The original contributions presented in the study are included in the article/supplementary material, further inquiries can be directed to the corresponding authors.

## Ethics statement

The studies involving human participants were reviewed and approved by Ruijin hospital affilited to Shanghai Jiaotong University. Written informed consent to participate in this study was provided by the participants’ legal guardian/next of kin.

## Author contributions

EC, YB, and HS preformed the conception design. PY, HS, and XZ drafted the manuscript. JL, JT, ZY, FJ, YC, YZ, and WQ conducted data extraction. ZX, XW, and CH analysed the data. XB, ZZ, and ZG revised the final manuscript. All authors contributed to the article and approved the submitted version.

## Conflict of interest

The authors declare that the research was conducted in the absence of any commercial or financial relationships that could be construed as a potential conflict of interest.

## Publisher’s note

All claims expressed in this article are solely those of the authors and do not necessarily represent those of their affiliated organizations, or those of the publisher, the editors and the reviewers. Any product that may be evaluated in this article, or claim that may be made by its manufacturer, is not guaranteed or endorsed by the publisher.
